# Recovery from opioid use on a neuropsychoanalytic service

**DOI:** 10.3389/fpsyt.2024.1409284

**Published:** 2024-06-19

**Authors:** Ross Meadon, Yanli Zhang-James, Sunny Aslam, Brian Johnson

**Affiliations:** ^1^ Norton School of Medicine, State University of New York (SUNY) Upstate Medical University, Syracuse, NY, United States; ^2^ Department of Psychiatry and Behavioral Sciences, State University of New York (SUNY) Upstate Medical University, Syracuse, NY, United States

**Keywords:** recovery from opioid use, drug-free treatment of opioid use, buprenorphine maintenance, 1+ year sobriety, chronic opioid therapy (COT), OUD treatment, effect of tobacco use on recovery from opioid use, detoxification from opioids

## Abstract

**Background:**

Little is known about recovery from opioid use disorder (OUD) or outcomes of detoxification and drug-free treatment of chronic opioid therapy (COT). Harm reduction with medications for opioid use disorder (MOUD) is regarded as the only legitimate treatment.

**Methods:**

The Institutional Review Board (IRB) approved reporting deidentified outcomes. Patients seen over a 10-year period whose records suggested recovery were called and interviewed.

**Results:**

Overall, 69/86 (80%) confirmed that they had been sober for at least a year, including 41 patients with OUD (75%) and 28 COT patients (90%). 91% were drug-free, and 9% were on MOUD. 79% preferred a psychotherapy approach. 21% preferred MOUD. Coming for more treatment and abstinence from tobacco were significantly correlated with recovery.

**Conclusion:**

This is the first report that we are aware of regarding the frequency of recovery from OUD and COT. We have complicated the discussion about what is the best treatment for patients with OUD and patients on COT. Advising that maintenance is the only legitimate treatment for patients who suffer from OUD or who are on COT seems both premature and jeopardizes the ability of treaters to individualize treatment recommendations.

## Introduction

Worldwide, 16 million individuals have had or currently suffer from opioid use disorder. In the United States in 2016, 11.5 million people misused opioid medications (1), Five to eight million were on chronic opioid therapy for pain (2), 3 million were addicted to opioids, and 0.5 million used heroin/illicit fentanyl (1). MOUD is often presented as the only option. Dr. Volkow, Director of the National Institute of Drug Abuse, has stated, “Detoxification (medically supervised withdrawal) is not recommended as OUD treatment, as most patients relapse, and their risk of overdose is particularly high due to reversal of tolerance.” (3).

Little is known about recovery from opioid use disorder. Hoffman, Vilsaint, and Kelly used the National Recovery Survey, an online survey that targeted a US noninstitutionalized population over 18 who had resolved a significant alcohol or drug problem, as indicated via an affirmative response to the screener question: “Did you used to have a problem with drugs or alcohol, but no longer do?” Among the sample of 2002 in recovery, they found 43 (2%) who reported 1–5 years of recovery from opioid addiction and 39 (2%) who reported more than 5 years of recovery. 9% of those with 1–5 years of recovery indicated that they were currently on buprenorphine maintenance. The use of buprenorphine for those with 5 or more years of recovery was not reported. The other two MOUDs, methadone and monthly intramuscular naltrexone, were not part of the recovery of persons who reported 1–5 years and were not reported for 5+ years of recovery. The drug-free portion of those in recovery 1–5 years was 91%. Hoffman, Vilsaint, and Kelly stated, “This study provides the first national estimate of opioid problem recovery prevalence and characterizes service utilization patterns and psychological well-being in a national probability-based sample of U.S. adults with opioid problem resolution.” (4). Of note, the small numbers of reported OUD recovery reveal nothing about how commonly there are recoveries of at least 1 year. We know what percentage of persons with OUD die per year, but not what percentage are able to enter sobriety. This is in contrast with alcohol use disorder (AUD) where a study of 4,422 subjects from the National Epidemiologic Study of Alcoholism and Related Conditions found the 1-year rate of recovery to be 36% with another 18% drinking without symptoms of alcoholism (5).

The only study of recovery from OUD, based on a small sample published in 2020, indicated that 91% achieved it drug free (4). The Director of the National Institute of Drug Abuse has said unequivocally that drug-free treatment is not to be carried out. Providers who treat a potentially lethal disease with 1.1% mortality per year on buprenorphine or methadone maintenance and 2.4% per year mortality with no treatment (6) may feel that if they provide the treatment that leads to recovery for the vast majority of OUD patients, they may be sued for malpractice when patients have adverse outcomes, outcomes that are guaranteed for some no matter what care is provided.

Practitioners may have the same fear about detoxifying patients on COT. Pain patients may have followed every rule their providers required when on COT, only to be told that their only alternative options are the three MOUD medications.

Narcotics Anonymous (NA) discourages maintenance therapy. The only requirement for NA membership is the desire to stop using. One maintenance program that studied 270 patients reported, “At 6-month follow-up, among participants who attended NA meetings within the past 3 months, over two-thirds reported that NA meetings were “quite a bit” or “extremely” helpful to their recovery (67%), whereas just 5% reported that NA meetings were “not at all helpful”. At 6 months, among participants who reported attending NA meetings while enrolled in buprenorphine maintenance treatment (BMT) (n = 209), only 33% reported disclosing their BMT status to an NA member. Of participants who disclosed their BMT status (n = 68), 26% reported that someone at NA encouraged them to stop taking buprenorphine or decrease their dose.” (7). One would think that NA group consciousness is our best source of information about how to recover from OUD (8). NA espouses drug-free recovery.

The neuropsychoanalytic approach to OUD and to chronic pain is unique in the following ways:

- New patients are required to arrive with a sober support person. This selects for persons who are willing to ask for help. It starts the process of constructing a recovery network. In many cases, it invites family members to witness the patient recounting their struggles with drugs as the history is taken and allows the treatment staff to explain to a family member how to be most effectively and lovingly helpful.
- Comprehensive evaluations determine all psychiatric, addiction, and pain diagnoses.
- OUD patients are offered their choice of detoxification and drug-free treatment, or buprenorphine maintenance. This decision is made in consultation with the support person and evaluators, trainees, and senior staff. They are told that whichever path they choose, the other approach is available on request.
- Medications to treat comorbid disorders are started immediately. Medications with addiction potential are never used. For example, frequently comorbid attention deficit hyperactivity disorder (ADHD) is treated with bupropion, desipramine, or atomoxetine.
- We understand that opioids help regulate human contact (9). Too much opioid tone hurts. For this reason, patients on buprenorphine do not want psychotherapy and cannot benefit from psychotherapy that requires closeness/therapeutic alliance (10). High opioid tone is a feature of autism that promotes autistic persons’ disengagement due to high C-terminal beta endorphin (11). We witness an autistic inability to tolerate contact when patients arrive on opioids and that it evaporates with detoxification, yielding an animated, related, engaged patient in place of the autistically distant one that we first met. The ability to feel emotions is restored.
- Detoxification involves arriving in early withdrawal, taking as much buprenorphine once that is needed to put withdrawal into remission, immediately starting low-dose naltrexone (LDN), which eases and shortens the withdrawal, being in transference focused psychotherapy daily during withdrawal, and twice a week thereafter for as long as the patient wishes, having medications for transient withdrawal symptoms: trazodone for insomnia, clonidine and olanzapine for anxiety, gabapentin for restless legs, hyoscyamine for gut cramps, and having unlimited cell phone access to senior staff. Detoxification is nearly 100% completed and so effective that senior staff are rarely called.
- Cell phone access begins the process of building a therapeutic alliance and constructing a holding environment. It conveys, “We are here for you.”
- We understand that opioid-induced hyperalgesia (OIH) is a nearly universal consequence of downregulating the endogenous opioid system with exogenous opioids. The degree of OIH is measured by the cold pressor test (CPT). When possible, the CPT is followed whereas LDN restores the receptor system by turning on opioid growth factor, requiring an average of three months (12).
- The goal of drug-free treatment is elimination of all addictive drugs, especially tobacco. Patients are told that there is only one craving pathway in the brain. Using any addictive drug will turn on craving for opioids. Varenicline is prescribed and taken whenever the patient feels nicotine craving.
- A frequent interpretation of avoidance is, “Most people with good recovery go to AA/NA, get to know people, and find a sponsor. You are not going. What is your thinking?” This makes our treatment a sophisticated form of 12 step facilitation. *We don’t instruct patients to attend AA. We ask them to work with us to consider unconscious reasons for their avoidance.* The monthly psychotherapy for buprenorphine maintenance also encourages 12 step meetings.
- New medical, physician assistant, or psychiatric nurse practitioner students, psychiatric residents, and addiction psychiatry fellows, are taught how to perform transference-focused psychotherapy with a combination of seminars, group supervision, and by having senior staff attend part of each psychotherapy hour, teaching in the room by showing how to interpret when the trainee misses an emotional association that requires a response.
- Senior staff are all prescribers, psychiatrists, or nurse practitioners. Pharmacologic management is part of every visit.
- By having senior staff see two to three patients per hour, whereas trainees provide 45-min psychotherapy, billing is robust enough to afford to treat mostly indigent Medicaid patients.

## Methods

Every new patient was asked to sign an informed consent form witnessed by the support person. Since we have a database of 1,663 patients seen from 2012 until the end of 2022, and IRB approval to follow outcomes, we were in a position to call our recovering patients to inquire about their experience. The following results shed more light on this question of recovery from OUD and chronic pain treated with opioid medications.

Electronic records were mined by using the names of the senior staff, the word naltrexone, and opioid use disorder. The senior author reviewed electronic records of 1,633 patients seen between 2012 and the end of 2022, initial intakes, and follow-up notes both from our service and other providers. Some patients initially evaluated on Addiction Medicine had had subsequent treatment on other services. The last treatment was recorded as the definitive treatment, drug free or MOUD. Patients were deemed relapsed if records documented addictive behaviors. Occasionally, the patient had not been seen after their initial evaluation. These patients were not counted as in recovery because recovery required at least 1 year of records after the initial evaluation. The number of visits was recorded. It was noted whether there was evidence of 1–5 years of recovery, and more than 5 years. Patients who had used MOUD and for whom there was no evidence of relapse were counted as recovering. Repeatedly obtaining opioids with pain complaints was counted as relapse. Being prescribed opioids briefly after an acute injury or surgery was not counted as a relapse. Being prescribed stimulants for ADHD or benzodiazepines was not counted as a relapse, whereas being seen in the emergency room for benzodiazepine withdrawal was.

The first author called every patient whose record review suggested they were in recovery. He used a standard interview, starting with whether there had been any relapse to drug use with clinically significant consequences. This meant that drinking alcohol without harm, inhaling either tobacco or cannabis, or using potentially addictive medications as prescribed was not counted as a relapse.

We used a linear regression model to compare the ages between the groups. Chi (2) tests were used to determine if the proportions of different sexes, diagnoses, uses of maintenance therapy or not, history of smoking, and the use of other addictive substances were significantly different between the recovery and relapsed groups. Finally, we used a logistic regression model to assess the association between these covariates and the status of recovery vs. relapse. We only included the covariates that were selected from an automated selection process implemented with the “GVSELECT” tool in STATA 18. We report the final adjusted odd ratios and their 95% confidence interval (95% CI).

## Results

There were 416 current and former patients identified who had no evidence of relapse. Of those, 378 had phone numbers in service. The first author tried each number at least twice, including in the evening or on the weekend. Of them, 114 picked up and 86 agreed to be interviewed.

Of the 86 patients interviewed, 50 (58.1%) were women and 36 (41.9%) were men. Of these patients, 54 had OUD (62.8%) and 32 (37.2%) were pain-only. The average age was 50.2 (±14.3), with pain-only patients being significantly older (58.1 ± 12.7) than those with OUD (45.6 ± 13.2, F_(1, 83)_ = 17.7, p = 0.0001).

Of the 86 patients, 53 had 1–5 years of being sober, and 33 had 5+ years of sobriety reported in their chart. At the interview, 69 (80%) confirmed that they had been sober for at least a year, including 39 patients with OUD (72.2%) and 30 patients with pain-only (93.8%), with a significantly higher proportion of pain-only patients who had been sober (χ^2^_(1)_ = 5.9, p = 0.02). The gender proportions and age were also similar between those recovering (age: 51.7 ± 14.9, 59% men) and relapsed patients (age: 44.4 ± 9.7, 53% men).

The proportions of recovering patients were similar for those who reported either 1–5 or 5+ years of sober per chart review (81.1% or 78.8%). Among them, the proportions of those who confirmed sobriety despite relapsing at least once were reported in **Table 1**. Only 11 of the 86 patients (12.8%) were on methadone or buprenorphine maintenance at the time of the call, whereas the majority were drug-free (87.2%). The proportions of the drug-free patients were similar between the recovering and relapsed patients (88.4% vs. 82.4%). The further breakdown of the drug-free vs. maintenance proportions by the number of years sober reported by chart and the sobriety status confirmed by interview is shown in **Table 1**. Overall, there were higher proportions of recovering patients who have been drug-free and no longer on maintenance therapy. Among those recovering patients, 23.3% of pain-only (7/30) and 35.9% OUD patients (14/39) had used potentially additive substances. These differences were not statistically significant.

**Table 1:** Composition of the recovery group among the 86 interviewed.

Subgroups	Total N of the subgroups (%)
Sober/patients with pain-only	30/32 (93.8%)
Sober/patients with OUD	39/54 (72.2%)
Sober/1–5 years sober	43/53 (81.1%)
Sober/5+ years sober	26/33 (78.8%)
Sober despite relapsing at least once/1–5 years sober	11/53 (20.8%)
Sober despite relapsing at least once/5+ years sober	7/33 (21.1%)
Drug–free/sober 1–5 years	37/43 (86.1%)
On maintenance/sober 1–5 years	6/43 (14.0%)
Drug-free/sober 5+ years	24/26 (92.3%)
On maintenance/sober 5+ years	2/6 (7.7%)
Using potentially addictive substances/sober pain-only	7/30 (23.3%)
Using potentially addictive substances/sober OUD	14/39 (35.9%)

All 32 pain-only patients had received neuropsychoanalytic care. Of them, 28 (87.5%) reported that neuropsychoanalytic care, phrased as either, “Dr. Johnson’s approach,” or, “the treatment you received at Addiction Medicine,” had been valuable. Of them, 27 remained sober at the time of the interview, with only 1 relapsed. Of the OUD patients, 40/54 (74.1%; 31 in recovery and 9 relapsed) reported the preference of the neuropsychoanalytic approach. Common comments about neuropsychoanalytic treatment were that it was provided with empathy, that each patient was treated as an individual, and “It saved my life.” Four out of 32 (12.5%) pain-only patients credited themselves for their recovery despite our treatment. Of the 54 patients with OUD, 14 (25.9%) reported that their recovery was attributed to methadone or buprenorphine maintenance is shown in **Table 2**.

**Table 2:** Preferred treatment.

	Preferred neuropsychoanalytic 68/86 (79.1%)	Preferred maintenance 18/86 (20.9%)
OUD (N = 54)	40 (74.1%)	14 (25.9%)
Sober (N = 39)	31 (79.5%)	8 (20.5%)
Relapsed (N = 15)	9 (60%)	6 (40%)
Pain-only (N = 32)	28 (87.5%)	4 (12.5%)
Sober (N = 30)	27 (90%)	3 (10%)
Relapsed (N = 2)	1 (50%)	1 (50%)

Recovering patients had come, on average, 18 times. The relapsed patients had come, on average, 14 times, significantly fewer than recovering patients (χ^2^_(1)_ = 17.9, p < 0.0001). Similar proportions of recovering patients (21/69, 30.4%) and relapsed patients (6/17, 35.3%) reported that they used potentially addictive drugs such as alcohol and marijuana without consequences. The proportions of those who used potentially additive substances in recovering pain-only or OUD patients were also similar (**Table 1**). 60.5% (52/86) of the interviewees reported smoking cigarettes at intake by the chart, with a significantly higher proportion of OUD patients (38/54, 70.4%) than of the pain-only patients (14/32, 44.8%) who smoked cigarettes previously (χ^2^_(1)_ = 6.0, p = 0.02). The baseline smoking rates were similar between those who recovered and those who relapsed. Only 15.1% (13/86) of the total interviewees were still smoking cigarettes at the time of the call with a significantly lower proportion of recovering patients (7/69, 10.1%) than relapsed patients (6/17, 35.3%) who were still smoking cigarettes (χ^2^_(1)_ = 6.7, p = 0.01) is shown in **Table 3**.

**Table 3:** Effects of smoking.

Subgroups	Current smoking rate
Relapsed	6/17 (35.3%)
In recovery	7/69 (10.4%)
Drug free	11/75 (14.7%)
Maintenance	2/11 (18.2%)

Among all the above variables examined, only the status of OUD or pain-only diagnosis and current cigarette smoking were selected to be included in the logistic regression model for the prediction of recovery vs. relapse. Smoking was associated with significantly increased odds of relapsing (adjusted OR 3.8, 95% CI: 1.02–14.0, χ^2^_(1)_ = 4.0, p = 0.046). The adjusted OR for pain-only vs. OUD diagnosis was not significant (0.2, 95% CI: 0.04–1.006).

Common comments about neuropsychoanalytic treatment were that it was provided with empathy, that each patient was treated as an individual, and “It saved my life.” Patients who were drug free but preferred maintenance said that the emotional intensity of psychotherapy was aversive.

## Discussion

This is the first paper to estimate the rate of recovery from OUD. We have found that recovery from OUD and treatment of chronic pain with opioid medications is unusual. If 80% of 416 patients had recovery of at least a year, that is one-fifth or 20% of the patients treated on Upstate Addiction Medicine. This suggests that recovery is less common than for alcohol use disorder, which is close to 50%.

The consensus among academics is that maintenance is the foremost recommendation. Experts in recovery from NA think the opposite. Our finding is similar to that of Hoffman, Vilsaint, and Kelly, the overwhelming majority of recovery is drug free. Our finding in a submitted paper, that the mortality for drug-free treatment of OUD and COT is lower than buprenorphine maintenance for patients that came for treatment more than ten times, suggests that drug free treatment is safe. (Assessing mortality for opioid use disorder: drug-free care v. buprenorphine maintenance) However, this finding in a clinical case series from a single clinic does nothing to protect practitioners from being sued for malpractice because of violating the community standard of care.

The goals of treatment are different. Drug-free treatment aims to result in persons who are free of all drugs, have all psychiatric disorders in remission, and who have mastered some of their childhood trauma. Among the responders, 44.8% of pain-only patients, and 70.4% of OUD patients, inhaled nicotine at the start of treatment, similar to the overall rates from our total 1,663 patients (72% of OUD patients and 44% of pain-only patients smoked cigarettes). (submitted papers: Assessing mortality for opioid use disorder: drug free care v. buprenorphine maintenance, Detoxification assisted with low dose naltrexone and psychotherapy for patients using chronic opioid therapy for pain significantly reduced mortality). Our finding that only 8/69 recovering interviewees were still using cigarettes suggests that we were mostly successful in putting tobacco use disorder into remission. Our finding of a significantly higher rate of smoking in relapsed patients reflects tobacco as a factor in relapse.

Limitations: This is a retrospective look at a clinical case series in a single clinic and therefore represents a convenience sample. It is mostly white/non-Hispanic, middle-aged, and more female. It is possible that we would not see significant differences across demographic groups because there was not much variation in our sample. Similarly, clinical characteristics may not vary because everyone was treated at one clinic. Generalizability and causal inferences are both inherently limited by the study design.

The size of our interviewed patient group is small. One aspect of treatment is that it requires active participation. The driver of OUD is being uncomfortable with feelings. Patient comments revealed that many, perhaps a majority of persons with OUD, would rather take drugs to get rid of their feelings, rather than tolerate the discomfort of psychotherapy.

## Conclusions

This is the first report that we are aware of regarding the frequency of recovery from OUD and COT. We have complicated the discussion about what is the best treatment for patients with OUD and patients on COT. Perhaps there is a way to use assessment to individualize treatment recommendations. If choosing the drug-free approach, patients must be warned that it requires a lot of treatment and tolerating emotional discomfort while working through issues. Advising that maintenance is the only legitimate treatment for patients who suffer from OUD or who are on COT seems both premature and jeopardizes the ability of treaters to individualize treatment recommendations.

## Data availability statement

The datasets presented in this article are not readily available because they contain privileged medical information. Requests to access the datasets should be directed to johnsonb@upstate.edu.

## Ethics statement

The studies involving humans were approved by SUNY Upstate institutional review board. The studies were conducted in accordance with the local legislation and institutional requirements. The participants provided their written informed consent to participate in this study. Written informed consent was obtained from the individual(s), and minor(s)’ legal guardian/next of kin, for the publication of any potentially identifiable images or data included in this article.

## Author contributions

RM: Data curation, Investigation, Writing – review & editing. BJ: Conceptualization, Data curation, Methodology, Project administration, Writing – original draft, Writing – review & editing. SA: Writing – review & editing. YZ-J: Conceptualization, Data curation, Methodology, Writing – review & editing.
